# Abdominal Actinomycosis Abscess Presenting as an Isolated Gastrointestinal Pseudotumor

**DOI:** 10.14309/crj.0000000000000672

**Published:** 2021-11-24

**Authors:** Danisa Clarrett, Jennifer Michelle Ray, Jason R. Taylor

**Affiliations:** 1 St. Francis Center for Digestive Disorders, St. Francis Hospital–Emory Healthcare, Columbus, GA; 2 Division of Gastroenterology and Hepatology, Department of Internal Medicine, Saint Louis University Hospital, Saint Louis, MO

## Abstract

*Actinomyces* is a gram-positive anaerobic bacterium that is ubiquitous in nature. It typically presents as respiratory, cervicofacial, or abdominopelvic abscesses. We present a 66-year-old man with a progressive enlarging abdominal wall nodule concerning for malignancy. The patient had a negative workup, including an ultrasound-guided fine-needle aspiration and colonoscopy, with biopsy for a possible extension to the colonic wall. Diagnosis of an *Actinomyces* abscess was obtained through surgical resection with right hemicolectomy. He was successfully treated with a prolonged course of intravenous antibiotics. This is a rare case of an isolated abdominal wall *Actinomyces* abscess mimicking a gastrointestinal malignancy.

## INTRODUCTION

Most *Actinomyces* species are commensal in the human oral, skin, gut, and vaginal flora.^1^ Approximately 20% of actinomycosis affects the abdomen. Although it can occur anywhere in the GI tract, the most common site is the proximal colon, particularly the appendix and ileocecal valve.^2,3^ It often forms a granulomatous nodule with sinus tracts but can also form cystic masses in the colon. Despite the presence of sinus tracts, these are rarely identified and extension to the abdominal wall is atypical.^2^ An isolated abdominal wall mass, as was seen in our patient, is an exceedingly unusual presentation of actinomycosis. This case report seeks to illustrate this unique manifestation of an uncommon infection.

## CASE REPORT

**Figure 1.**
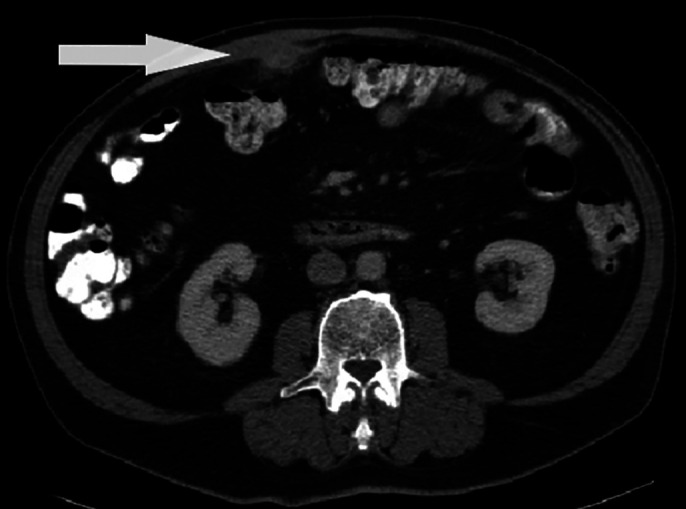
Initial abdominal and pelvic computed tomography with contrast showing an ill-defined round density mass (white arrow) measuring 2.5 cm in greatest diameter adherent to or arising from the posterior margin of the abdominal wall.

The patient is a 66-year-old man with a history of celiac sprue who presented to the gastroenterology clinic to evaluate a new abdominal wall nodule. The patient was otherwise asymptomatic. Abdominal and pelvic computed tomography showed an ill-defined, round density mass measuring approximately 2.5 cm in greatest diameter, just to the right of midline, with surrounding fat stranding (Figure 1). This was monitored clinically.

**Figure 2.**
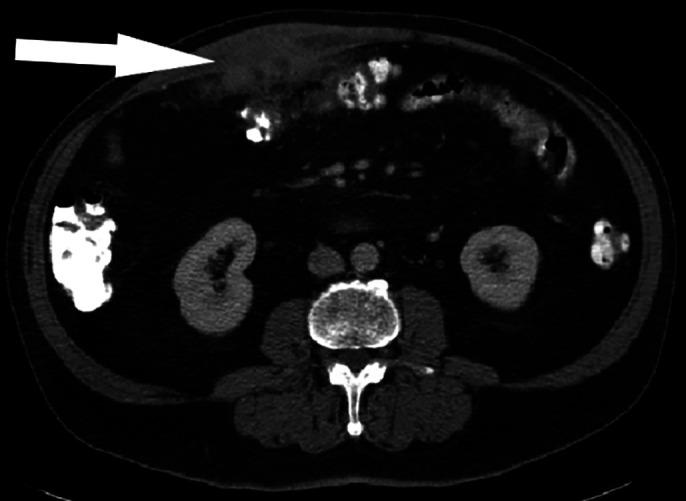
Abdominal and pelvic computed tomography with contrast 6 months after initial presentation showing increased size of soft-tissue density with surrounding fat stranding within the right medial rectus measuring 6.2 × 2.2 cm (white arrow).

Surveillance imaging performed 6 months later showed that the mass had doubled in size (Figure 2). The mass was adjacent to the transverse colon with noted mesenteric inflammation; however, there was no obvious mucosal invasion. On physical examination, he had an 8 × 10-cm mobile, nontender right upper quadrant abdominal wall mass. Ultrasound-guided fine-needle aspiration (FNA) showed acute and chronic inflammation with no evidence of malignancy.

**Figure 3.**
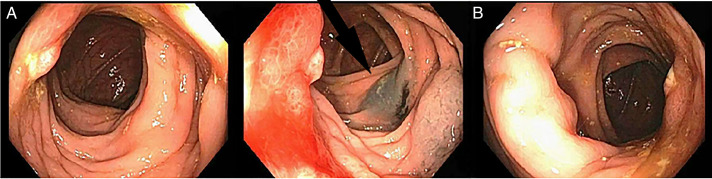
Diagnostic colonoscopy showing multiple views of localized erythematous, nodular, scarred mucosa in the transverse colon with proximally placed tattoo spot ink (black arrow).

Owing to the lesion's proximity to the colon and concern for underlying malignancy or inflammatory bowel disease, the patient underwent a diagnostic colonoscopy. This showed scarred, erythematous, and nodular mucosa in the transverse colon (Figure 3). An India ink tattoo was placed proximally and distally for possible surgical resection. Biopsies of this area revealed ulceration with histiocytic and neutrophilic infiltrates, no malignancy, and negative acid-fast bacillus and Gram stains. The patient had no systemic symptoms or change in bowel habits at this time.

Given the nondiagnostic results of these studies, the patient went for surgical resection of the right upper abdominal wall mass en bloc with right hemicolectomy to resect the affected mid and proximal transverse colon. Surgical pathology of the abdominal wall mass revealed actinomycosis with abscess formation but was negative for dysplasia or malignancy. The colon pathology was negative except for mild histiocytic and neutrophilic infiltration. The patient completed a 6-week course of IV penicillin. On the 1-year follow-up, the patient was doing well, with no evidence of recurrence and healthy ileocolonic anastomosis on follow-up diagnostic colonoscopy.

## DISCUSSION

Abdominal actinomycosis can present with a wide variety of nonspecific symptoms (ie, fever, anorexia, and diarrhea), but it most frequently presents with abdominal tenderness.^4^ Our patient had a nontender abdominal wall nodule and was otherwise asymptomatic. Owing to its occasional mass-like formation and size progression, as was seen in our patient, abdominal actinomycosis can be easily mistaken for a malignant process.^5^

Computed tomography is often the first diagnostic approach in abdominal actinomycosis because it may help to characterize the size and extent of disease; however, it is unlikely to differentiate other diagnoses, such as Crohn's disease, abdominal tuberculosis, or malignancy.^5^ For this reason, FNA is considered the gold standard for diagnosis. A diagnostic aspiration would classically display filamentous bacteria with sulfur granules and a positive periodic acid-Schiff reaction. Of note, FNAs frequently have false-negative results.

Concerns for underlying malignancy frequently prompt endoscopic evaluation.^6^ In our patient, erythematous, nodular, and scarred mucosa was visualized and biopsied in the transverse colon. Other case reports demonstrate findings of colonic masses. Even in the case of significant mucosal abnormalities, biopsy results are often nonspecific, showing chronic inflammation.^7^

Many cases of abdominal actinomycosis will require a surgical approach for definitive diagnosis.^4^ The diagnosis is made preoperatively in only 17%–20% of cases. Given its great response to antibiotics, it has been suggested that surgery is reserved only in cases where a diagnosis has not yet been revealed.^8^ Based on literature review, there are only 29 cases of anterior abdominal wall actinomycosis reported as of 2012.^9^

The actinomycetes are almost uniformly susceptible to β-lactam antibiotics. It is recommended to treat with intravenous penicillin G for 4–6 weeks, followed by oral penicillin V for up to a year.^3^ Studies have shown much lower susceptibility to metronidazole and fluoroquinolones.^3,10^ It is important to remember the polymicrobial nature of many *Actinomyces* infections. It is often found in conjunction with more resistant organisms, and this can complicate treatment regimens.

Abdominal actinomycosis should always be considered in the differential diagnosis for an abdominal wall mass if the initial workup is negative. A higher level of suspicion is especially warranted in those with chronic illness and immunocompromised states, such as in our older patient with celiac sprue. Earlier consideration of this diagnosis paired with improved diagnostic tests can reduce the need for surgery.^8^ When a diagnosis is finally achieved, treatment is usually complete, and the prognosis is excellent. Our case highlights a rare presentation of abdominal actinomycosis.

## DISCLOSURES

Author contributions: All authors contributed equally to this article. J. Ray is the article guarantor.

Financial disclosure: None to report.

Informed consent was obtained for this case report.
